# Glycosaminoglycans and fucoidan have a protective effect on experimental glomerulonephritis

**DOI:** 10.3389/fmolb.2023.1223972

**Published:** 2023-07-05

**Authors:** Baranca Buijsers, Marissa Maciej-Hulme, Maaike Jacobs, Marinka Bakker-van Bebber, Mark de Graaf, Rustem Salmenov, Naomi Parr, Ton J. Rabelink, Tom Nijenhuis, Johan van der Vlag

**Affiliations:** ^1^ Department of Nephrology, Radboud Institute of Molecular Life Sciences, Radboud University Medical Center, Nijmegen, Netherlands; ^2^ Division of Nephrology, Department of Internal Medicine, The Einthoven Laboratory for Vascular and Regenerative Medicine, Leiden University Medical Center, Leiden, Netherlands

**Keywords:** glycosaminoglycans, heparan sulfate, fucoidan, sulodexide, endothelial glycocalyx, heparanase-1, proteinuria, glomerulonephritis

## Abstract

**Background:** The glomerular endothelial glycocalyx is degraded during inflammation. The glycocalyx plays a pivotal role in endothelial function and is involved in many processes including binding of chemokines and cytokines, leukocyte trafficking, and preventing proteinuria. HS-based therapeutics are a promising novel class of anti-inflammatory drugs to restore a compromised endothelial glycocalyx under inflammatory conditions. Recently, we demonstrated that treatment with HS extracted from unstimulated glomerular endothelial glycocalyx (unstimulated HS_glx_) reduced albuminuria during anti-GBM induced glomerulonephritis. Since endothelial HS domains are distinct in unstimulated *versus* inflammatory conditions, we hypothesized that 1) unstimulated HS_glx_, 2) LPS-stimulated HS_glx_, 3) the HS-mimetic fucoidan and 4) the glycosaminoglycan preparation sulodexide, which is a mixture of low molecular weight heparin and dermatan sulfate, might have different beneficial effects in experimental glomerulonephritis.

**Methods:** The effect of unstimulated HS_glx_, LPS HS_glx_, Laminaria japonica fucoidan, or sulodexide on experimental glomerulonephritis was tested in LPS-induced glomerulonephritis in mice. Analyses included urinary albumin creatinine measurement, cytokine expression in plasma and renal cortex, and renal influx of immune cells determined by flow cytometry and immunofluorescence staining. Furthermore, the observed *in vivo* effects were evaluated in cultured glomerular endothelial cells and peripheral blood mononuclear cells by measuring cytokine and ICAM-1 expression levels. The ability of the compounds to inhibit heparanase activity was assessed in a heparanase activity assay.

**Results:** Treatment of mice with LPS HS_glx_ or sulodexide near-significantly attenuated LPS-induced proteinuria. All treatments reduced plasma MCP-1 levels, whereas only fucoidan reduced IL-6 and IL-10 plasma levels. Moreover, all treatments reversed cortical ICAM-1 mRNA expression and both fucoidan and sulodexide reversed cortical IL-6 and nephrin mRNA expression. Sulodexide decreased renal influx of CD45^+^ immune cells whereas renal influx of macrophages and granulocytes remained unaltered for all treatments. Although all compounds inhibited HPSE activity, fucoidan and sulodexide were the most potent inhibitors. Notably, fucoidan and sulodexide decreased LPS-induced mRNA expression of ICAM-1 and IL-6 by cultured glomerular endothelial cells.

**Conclusion:** Our data show a potentially protective effect of glycosaminoglycans and fucoidan in experimental glomerulonephritis. Future research should be aimed at the further identification of defined HS structures that have therapeutic potential in the treatment of glomerular diseases.

## Introduction

Glomerulonephritis is characterized by immune-mediated glomerular damage in primary glomerular disease such as anti-glomerular basement membrane disease or secondary glomerular disease like post-infectious glomerulonephritis induced kidney failure (Tecklenborg et al., 2018). Glomerulonephritis is characterized by glomerular influx of leukocytes, albuminuria and loss of kidney function, which may ultimately proceed to end-stage renal disease (Duffield, 2010).

The endothelial glycocalyx is a thick glycan layer that lines blood vessels, contributes to charge and size-selective blood filtration and plays a central role in inflammation. A large proportion of the endothelial glycocalyx is comprised of non-sulfated hyaluronic acid, which provides the gel-like structure of the glycocalyx. Heparan sulfate (HS) is a linear, negatively charged glycosaminoglycan (GAG) that provides specificity to the endothelial glycocalyx function by binding of chemokines and cytokines, adherence of leukocytes, and contributes to the size and charge selective barrier function of the endothelium (Fridén et al., 2011; Garsen et al., 2014; van der Vlag and Buijsers, 2020). The diverse functions of HS are dictated by its enormous structural complexity, which leads to an immense number of structurally different domains that can bind specific ligands and thus modulates multitude physiological and pathological processes (Kreuger and Kjellén, 2012).

The HS-modifying enzymes are differentially expressed in tissues, cell types and under, for example, inflammatory conditions, thereby creating tissue-, cell- and context-specific expression of HS domains (Saphire et al., 2001; Carter et al., 2003; Rops et al., 2004a). Previously, we identified specific HS domains in the glomerular endothelial glycocalyx that are involved in binding of leukocytes, which was associated with an altered expression of HS-modifying enzymes (Rops et al., 2007a; Rops et al., 2008). Under healthy conditions, endothelial HS prevents the adhesion of inflammatory components, such as selectins and integrins expressed by leukocytes, as well as cytokines and chemokines. However, extensive remodelling of HS occurs during inflammation and results in the display of pro-inflammatory HS domains (Rops et al., 2007b; Rops et al., 2008; Farrugia et al., 2018). These pro-inflammatory HS domains can bind chemokines that attract and activate leukocytes, as well as selectins and integrins expressed by leukocytes. This pro-inflammatory attraction and activation contributes to the infiltration of leukocytes that release tissue-damaging effector molecules.

HS in the endothelial glycocalyx can be degraded by heparanase-1 (HPSE) under inflammatory conditions. HPSE is the only known mammalian enzyme that can cleave HS. HPSE can degrade HS domains, thereby allowing the synthesis of new HS domains that, for example, can bind chemokines, selectins and integrins (Garsen et al., 2014; van der Vlag and Buijsers, 2020). The expression of HPSE is increased in the majority of glomerular diseases and linked to a reduced HS expression and the development of proteinuria. Notably, we recently showed that HPSE is essential for the development of proteinuria and renal damage in experimental glomerulonephritis and diabetic nephropathy, since HPSE-deficient mice displayed reduced proteinuria compared to wild-type mice (Gil et al., 2012; Garsen et al., 2016).

The importance of HPSE in the development of proteinuria implies that compounds that inhibit HPSE activity may have therapeutic benefits for patients with inflammatory glomerular diseases (Masola et al., 2018; van der Vlag and Buijsers, 2020). Drugs that inhibit HPSE can be divided into two groups. Firstly, inhibitors that directly block the HS-binding and active site of HPSE. Secondly, HS-mimetics, which are drugs that structurally resemble HS such as heparin-based drugs (Farrugia et al., 2018; van der Vlag and Buijsers, 2020). Currently, a few heparin-based HPSE inhibitors are under development. The heparin-based HS mimetic SST0001 has for instance been shown to reduce albuminuria 2-fold in experimental type 1 and 2 diabetic nephropathy (Ritchie et al., 2011; Gil et al., 2012). Another GAG preparation that has been explored as possible therapeutic in glomerular disease is sulodexide, which is a mixture of low molecular weight heparin (LMWH) and dermatan sulfate. Conflicting results have been obtained with regard to sulodexide. Sulodexide effectively restored the glycocalyx thickness and showed a trend towards normalization of systemic albumin clearance in a study of type 2 diabetes mellitus patients, whereas no such effect was observed in two other studies (Poplawska et al., 1997; Broekhuizen et al., 2010; Lewis et al., 2011; Packham et al., 2012; Rabelink et al., 2017). These contradicting findings can possibly be explained by lack of insight into the specific structures within different sulodexide preparations due to different animal sources used for isolation.

Fucoidans are a class of heterogenous, branched, sulfated polysaccharides that are rich in fucose residues. Fucoidans can be considered as HS-mimetics and might therefore inhibit HPSE activity as well. In particular, fucoidan extracted from the brown seaweed Laminaria japonica resembles HS due to the presence of sulfate groups at similar positions (C-2 and C-3) on the sugar residues and some alpha-1,4 glycosidic linkages in the backbone of the polysaccharide (Wang et al., 2010; Yuan et al., 2022). Due to the low toxicity and wide availability, fucoidans, from a variety of sources, have been investigated as novel carbohydrate-based drugs in cancer, acute pancreatitis, COVID-19, and various kidney diseases (Carvalho et al., 2014; van Weelden et al., 2019; Wang et al., 2022; Yuan et al., 2022; Zahan et al., 2022).

In a recent study, we demonstrated that treatment with exogenous HS extracted from glycocalyx of non-activated glomerular endothelial cells (unstimulated HS_glx_) attenuates albuminuria in anti-glomerular basement membrane (GBM) induced glomerulonephritis (Maciej-Hulme, van Gemst et al., recently accepted in Frontiers). Since endothelial HS domains are distinct in unstimulated *versus* inflammatory conditions, we hypothesize that HS extracted under different conditions and compounds similar to HS might have diverse beneficial effects in experimental glomerulonephritis. Therefore, we investigated the effect of 1) unstimulated HS_glx_, 2) HS-derived from activated glomerular endothelial cells (LPS HS_glx_), 3) the GAG related HS-mimetic fucoidan and 4) the GAG sulodexide, which is a mixture of low molecular weight heparin (LMWH) and dermatan sulfate, on the clinical outcome of experimental glomerulonephritis in mice. Furthermore, we explored the ability of these four compounds to inhibit HPSE activity.

## Methods

### Animals

Eight-to-ten-week-old C57Bl/6J female mice (Charles River, Cologne, Germany) were kept under specific pathogen-free conditions and housed in a temperature-controlled room with a 12-h light/dark cycle with *ad libitum* access to food and water. All experiments were approved by the Animal Ethical Committee of the Radboud University Nijmegen and the Dutch government.

### Experimental disease models

Experimental LPS-induced glomerulonephritis was induced in ten-week-old wt C57Bl/6J mice by an i.p. Injection with 110 µg LPS (O111:B4; Sigma-Aldrich, Zwijndrecht, Netherlands) as described previously (Garsen et al., 2016). The mice were treated with 11 µg of HS extracted from unstimulated endothelial glycocalyx (Unstimulated HS_glx_), HS extracted from LPS stimulated endothelial glycocalyx (LPS HS_glx_), fucoidan extracted from Laminaria japonica (kind gift of Dr. Bob Long, MicroVascular Health Solutions LLC, Alpine, UT) or sulodexide (kind gift of Dr. Enrique Poradosu, Keryx Biopharmaceuticals, Boston, United States) in PBS via i. v. Injection. I.v. Injection with the treatment compounds was performed prior to i.p LPS injection. Mice were sacrificed 48 h after LPS injection.

Kidneys were collected and snap frozen in liquid nitrogen and urine was collected after 18 h housing in metabolic cages upon termination. Urinary albumin was measured by radial immunodiffusion (Mancini) as described previously (Garsen et al., 2016). Blood urea nitrogen (BUN) and urinary or plasma creatinine concentrations were determined routinely in our clinical diagnostic facility.

### Cell culture

Mouse glomerular endothelial cells (mGEnC-1) were cultured as described previously (Rops et al., 2004b). Differentiated mGEnC-1 were stimulated with 100 ng/mL LPS (O111:B4; Sigma-Aldrich) and treated with 10 μg/mL of unstimulated HS_glx_, LPS HS_glx_, Fucoidan or sulodexide. Treatment was added 1 h prior to LPS stimulation.

Human peripheral blood mononuclear cells (PBMCs) were isolated by differential centrifugation over Ficoll-Paque (Lymphoprep, StemCell Technologies, Inc.). PBMCs were washed three times in PBS, resuspended in RPMI culture medium supplemented with 2 mM glutamax, 1 mM pyruvate and penicillin/streptomycin (all from Thermo Fisher Scientific, Breda, Netherlands), and counted on a Casy counter. PBMCs from at least three different donors were seeded into 96-well flat bottom plates in a density of 500,000 cells per well. Cells were allowed to adhere for 1 h at 37°C. Cells were washed three times with PBS prior to stimulations. PBMCs were stimulated with 10 ng/mL LPS (*E. coli* 055:B5; Cat#trlrl-pb5lps; Invivogen, Huissen, Netherlands) and 10 μg/mL of unstimulated HS_glx_, LPS HS_glx_, fucoidan or sulodexide. Treatment was added 1 h prior to LPS stimulation.

### Extraction, isolation and fractionation of glycocalyx constituents

Glycocalyx was extracted from mGEnC cell layers by overnight digestion with 125 μg/mL proteinase K (Merck chemicals B.V., Amsterdam, Netherlands) in 50 mM Tris-HCl (pH: 7.9), 10 mM NaCl, 3 mM MgCl2, 1% triton X-100 buffer, followed by overnight DNAse-I (Qiagen, Venlo, Netherlands) and RNAse (GE-healthcare, Eindhoven, Netherlands) treatment at 37°C. NaCl was added to digested extracts (final concentration of 2 M), followed by chloroform (1:1), vortexing and centrifugation for 20 min at 4,636 g to separate the phases. The upper layer (aqueous phase) was dialyzed against 5 × 5 L baths of Milli-Q H2O using SnakeSkin dialysis membranes (MWCO 3500 Da, Thermo Fisher Scientific) and dried using a Savant SC210A Speed-Vac concentrator (Thermo Fisher Scientific). Isolation of HS_glx_ from extracted glycocalyx was performed as described previously (Guimond et al., 2009; Maciej-Hulme et al., 2020). In brief, dialyzed and concentrated glycocalyx extracts were digested with 125 mU of Chondroitinase ABC (Sigma-Aldrich) in 25 mM Tris, 2 mM Mg(Ac)_2_ pH 8 for 18 h before fractionation by anion exchange chromatography using DEAE-sepharose CL-6B beads (Sigma-Aldrich) equilibrated in PBS. Bound HS_glx_ was washed with 0.25 M NaCl in PBS, pH 7.4 and then eluted with 2 M NaCl in PBS, pH 7.4. Isolated HS_glx_ was desalted via PD10 desalting columns (GE Healthcare, Sephadex G25) using Milli-Q H_2_O. Size fractionation of purified mGEnC HS_glx_ was performed in 0.25 M ammonium bicarbonate at 0.22 mL/min using a BioGel P10 resin column (75 mm × 16 mm, 90–180 µm beads, Bio-Rad, Lunteren, Netherlands). 1 mL fractions were collected and pooled into corresponding peaks. Pooled fractions were dialyzed against Milli-Q water and dried. Mass spectrometry was performed as previously described (Maciej-Hulme, van Gemst et al., recently accepted in frontiers) and Fraction 2 (F2) was selected based on activity in previous experiments (Maciej-Hulme, van Gemst et al., recently accepted in frontiers) as well as the small size of these oligosaccharide structures (between tetra- and hexasaccharides).

### Cytokine measurements

Cytokine levels in plasma from mice were measured using the LEGENDplex mouse inflammation panel (Cat# 740150; Biolegend, Amsterdam, Netherlands). The cytokines included in this panel are IL-1α, IL-1β, IL-6, IL-10, IL-12p70, IL-17A, IL-23, IL-27, MCP-1, IFN-β, IFN-γ, TNF-α, and GM-CSF. The experiment was performed according to manufacturer’s instructions and data was analyzed using the provided software.

Cytokine production of mGEnC-1 and PBMCs was measured in culture supernatants using the commercial ELISA kits mouse DuoSet MCP-1 (Cat#DY479; R&D systems, Bio-techne, Abingdon, United Kingdom), human DuoSet IL-6 (Cat#DY206; R&D systems), human DuoSet TNF-α (Cat#DY210; R&D systems), and human DuoSet IL-10 (Cat#DY217; R&D systems). The entire procedure was performed according to manufacturer’s instructions. Samples were diluted 20 times in PBA (1% BSA in PBS) for PBMC culture supernatants and 5 times in PBA for mGEnC-1 culture supernatants.

### RNA isolation and real-time PCR

RNA was isolated from the kidney cortex using RNeasy mini kit (Qiagen) and from mGEnC-1 using TRIzol (Thermo Fisher Scientific) and 1 µg of RNA was reverse transcribed into cDNA using the Transcription First Strand cDNA synthesis kit (Roche, Woerden, Netherlands) according to manufacturer’s instructions. Quantitative PCR was performed with SYBR Green (Roche) on a CFX 96 C1000 Thermal Cycler (Bio-rad). Data was analyzed using glyceraldehyde-3-phosphate dehydrogenase (GAPDH) as housekeeping gene and by making use of the delta-delta CT method. Sequences of gene-specific primers are listed in Table 1.

**TABLE 1:** Primers used in real-time PCR.

Target	Primer sequence
GAPDH	F: 5′-AGA​AAC​CTG​CCA​AGT​ATG​ATG​AC-3′
	R: 5′-TCA​TTG​TCA​TAC​CAG​GAA​ATG​AG-3′
MCP-1	F: 5′-TGA​TCC​CAA​TGA​GTA​GGC​TGG​AG-3′
	R: 5′-ATG​TCT​GGA​CCC​ATT​CCT​TCT​TG-3′
IL-6	F: 5′-GAG​GAT​ACC​ACT​CCC​AAC​AGA​CC-3′
	R: 5′-AAG​TGC​ATC​ATC​GTT​GTT​CAT​ACA-3′
IL-10	F: 5′-GTG​GAG​CAG​GTG​AAG​AGT​GA-3′
	R: 5′-TGC​AGT​TGA​TGA​AGA​TGT-3′
ICAM-1	F: 5′-GTC​GAA​GGT​GGT​TCT​TCT​GAG-3′
	R: 5′-TCC​GTC​TGC​AGG​TCA​TCT​TAG​G-3′
VCAM-1	F: 5′-CAT​GAA​CAG​ACA​GGA​GTT​TTC​TTC​A-3′
	R: 5′-ATT​TAG​CTC​GGC​AAA​CAA​GAG​C-3′
Nephrin	F: 5′-CCC​TCC​AGT​TAA​CTT​GTC​TTT​G-3′
	R: 5′-ATG​CAG​CGG​AGC​CTT​TGA-3′
HPSE	F: 5′-GAG​CGG​AGC​AAA​CTC​CGA​GTG​TAT​C-3′
	R: 5′-GAT​CCA​GAA​TTT​GAC​CGT​TCA​GTT​GG-3′
TNF-α	F: 5′-CTG​TAG​CCC​ACG​TCG​TAG​C-3′
	R: 5′-TTG​AGA​TCC​ATG​CCG​TTG-3′
Desmin	F: 5′-CAG​TCC​TAC​ACC​TGC​GA-3′
	R: 5′-GCC​ATC​TTC​ACA​TTG​AGC​AGG-3′
eNOS	F: 5′-GGT​AGT​TAG​GGC​ATC​CTG​CTG-3′
	R: 5′-GTC​TGG​GAC​TCA​CTG​TCA​AAG-3′

F, forward; R, reverse; GAPDH, glyceraldehyde-3-phosphate dehydrogenase; MCP-1, monocyte chemoattractant protein-1; ICAM-1, intercellular adhesion molecule 1; VCAM-1, vascular cell adhesion molecule-1; HPSE, heparanase; TNF-α, tumor necrosis factor-alpha; eNOS, endothelial nitric oxide synthase.

### Immunofluorescence staining

Glomerular presence of granulocytes and monocytes, and expression of ICAM-1, VCAM-1, agrin (marker for glomerular basement membrane (GBM)), PDGFR (marker for mesangial cells), and CD31 (marker for endothelial cells) was determined by immunofluorescence staining on 2 µm sections of fresh-frozen kidneys. Primary antibodies included rat anti-mouse Gr-1 (Ly6G) (cat#14-5931-85, Invitrogen, RRID:AB_467731), rat anti-mouse CD68 (Cat#MCA 1957, RRID:AB_322219 Serotec, Oxford, United Kingdom), rat anti-mouse ICAM-1 (Cat# 14-0541-85, RRID:AB_467302, Thermo Fisher Scientific), rabbit anti-mouse VCAM-1 (Cat# NBP2-67292, Novus Biologicals), Armenian hamster anti-mouse MI-91 (anti-agrin (Raats et al., 1998)), rabbit anti-mouse PDGFR (Cat# ab32570, RRID:AB_777165, Abcam, Cambridge, United Kingdom), and rat anti-mouse CD31 (Cat# 553369, RRID:AB_394815, BD Biosciences, Vianen, Netherlands). Secondary antibodies included goat anti-rat alexa 488 (Cat# A-11006, RRID:AB_2534074, Thermo Fisher Scientific) for Gr-1, CD68 and ICAM-1, chicken anti-rabbit alexa 488 (Cat# A-21441, RRID:AB_2535859, Thermo Fisher Scientific) for VCAM-1 and PDGFR, goat anti-rat alexa 594 (Cat#A-11007, RRID:AB_10561522, Thermo Fisher Scientific) for CD31, and goat anti-armenian hamster 594 (Cat#A78966, Thermo Fisher Scientific) for MI-91. Quantification of granulocytes and monocytes was performed by counting the number of granulocytes and monocytes in 50 glomeruli per section. Staining intensity of ICAM-1 was scored in 30 glomeruli per section on a scale between 0 (no staining) and 10 (maximal staining). Counting and scoring was performed on blinded sections by two independent investigators.

### Flow cytometry

Half of a kidney, directly obtained after sacrificing, was used for flow cytometry analysis. Kidneys were cut in pieces and put in an enzymatic digestion solution containing 0.2 mg/mL Collagenase IV (Cat#C5138, Sigma-Aldrich) and 0.05 mg/mL deoxyribonuclease (DNase) I (Cat#1 0104159 001, Sigma-Aldrich), and placed in a 37°C incubator for 60 min. Cells were forced through a 70 μm strainer and resuspended in an Ammonium-Chloride-Potassium (ACK) lysis buffer (155 mM NH4Cl, 10 mM KHCO3, 0.1 mM EDTA) for 5 min at room temperature (RT). For flow cytometry staining, 5 × 10^6^ cells were used per sample. Possible Fc receptors on cells were blocked with mouse BD FC block (Cat#553142, BD Bioscience). The antibodies that were used in this study are described in Table 2. The antibody dilutions ranged from 1:200 to 1:100. Flow cytometry was performed with the NovoSampler Pro (ACEA Biosciences, San Diego, United States), and data were analyzed in the NovoExpress software version 1.5.6 (ACEA Biosciences) as described in Supplementary Figure S4.

**TABLE 2:** Antibodies used for flow cytometry panel to identify myeloid cells in the kidney.

Antibody	Fluorophore	Target	Company	RRID
anti-CD45	Brilliant Violet 510	Leukocytes	Cat#103137 Biolegend	AB_2561392
anti-Ly-6C	Brilliant Violet 785	Monocytes	Cat#128041 Biolegend	AB_2565852
anti-CD11b	APC/Cyanine7	Monocytes Dendritic cells Granulocytes	Cat#101225 Biolegend	AB_830641
anti-CD11c	APC	Dendritic cells	Cat#117309 Biolegend	AB_313778
anti- Ly-6G	Brilliant Violet 650	Granulocytes	Cat#127641 Biolegend	AB_2565881
anti-CD49b	eFluor450	Lineage NK and T cells	Cat#48-5971-82 Thermo Fisher Scientific	AB_10671541
anti-CD90.2	eFluor450	Lineage T cells	Cat#48-0902-82 Thermo Fisher Scientific	AB_1272200
anti-Nk1.1	eFluor450	Lineage NK and T cells	Cat#48-5941-82 Thermo Fisher Scientific	AB_2043877
anti-Ter119	eFluor450	Lineage erythroid cells	Cat#48-5921-82 Thermo Fisher Scientific	AB_1518808
anti-CD45R	eFluor450	Lineage B cells	Cat#48-0452-82 Thermo Fisher Scientific	AB_1548761

Lineage represents a cocktail of antibodies with the same fluorophore.

### HPSE activity assay

The inhibition of HPSE activity by unstimulated HS_glx_, LPS HS_glx_, fucoidan or sulodexide was determined by an in-house developed HPSE activity assay, which was validated by the use of recombinant active human HPSE (Cat#7570-GH-005, Bio-techne), and performed as described previously (Buijsers et al., 2020a). Briefly, Nunc maxisorp flat bottom 96 plates (Thermo Scientific, Breda, Netherlands) were coated with 10 μg/mL heparan sulfate from bovine kidney (HSBK) (Sigma-Aldrich) in HS coating buffer, overnight in a humidified chamber at RT. Subsequently, plates were blocked for minimal 2 h with 1% bacto-gelatin (Difco laboratories, Detroit, Michigan, United States) in PBS at RT, whereupon HPSE inhibitors were added to the plate with a standard amount of recombinant HPSE in HPSE buffer. Remaining HSBK was detected with primary mouse anti-rat IgM HS antibody JM403 (Cat#370730-S, RRID: AB_10890960, 1 μg/mL in PBST, Amsbio, Abingdon, United Kingdom) for 1 h at RT. Subsequently, plates were incubated with secondary goat anti-mouse IgM HRP antibody (Cat#1020-05, RRID: AB_2794201, dilution 1:10,000 in PBST, Southern Biotech, Uden, Netherlands) for 1 h at RT. Finally, 3,3′,5,5′-tetramethylbenzidine (TMB) substrate (Invitrogen) was added and the reaction was stopped by addition of 2 M sulfuric acid, and absorbance was measured at 450 nm.

### Statistical analyses

Values are expressed as mean ± SEM. Shapiro-Wilk test or Kolmogorov-Smirnov test was performed to test for normality of data. Significance was determined by one-way ANOVA followed by Dunnett’s test or Kruskal–Wallis test followed by Dunn’s test to compare more than two groups. All analyses were performed using GraphPad Prism V.9.1.2 (La Jolla, CA, United States). *p* values less than 0.05 were considered as statistically significant.

## Results

### Treatment with LPS HS_glx_ or sulodexide tend to reduce proteinuria in mice with experimental glomerulonephritis

**FIGURE 1:**
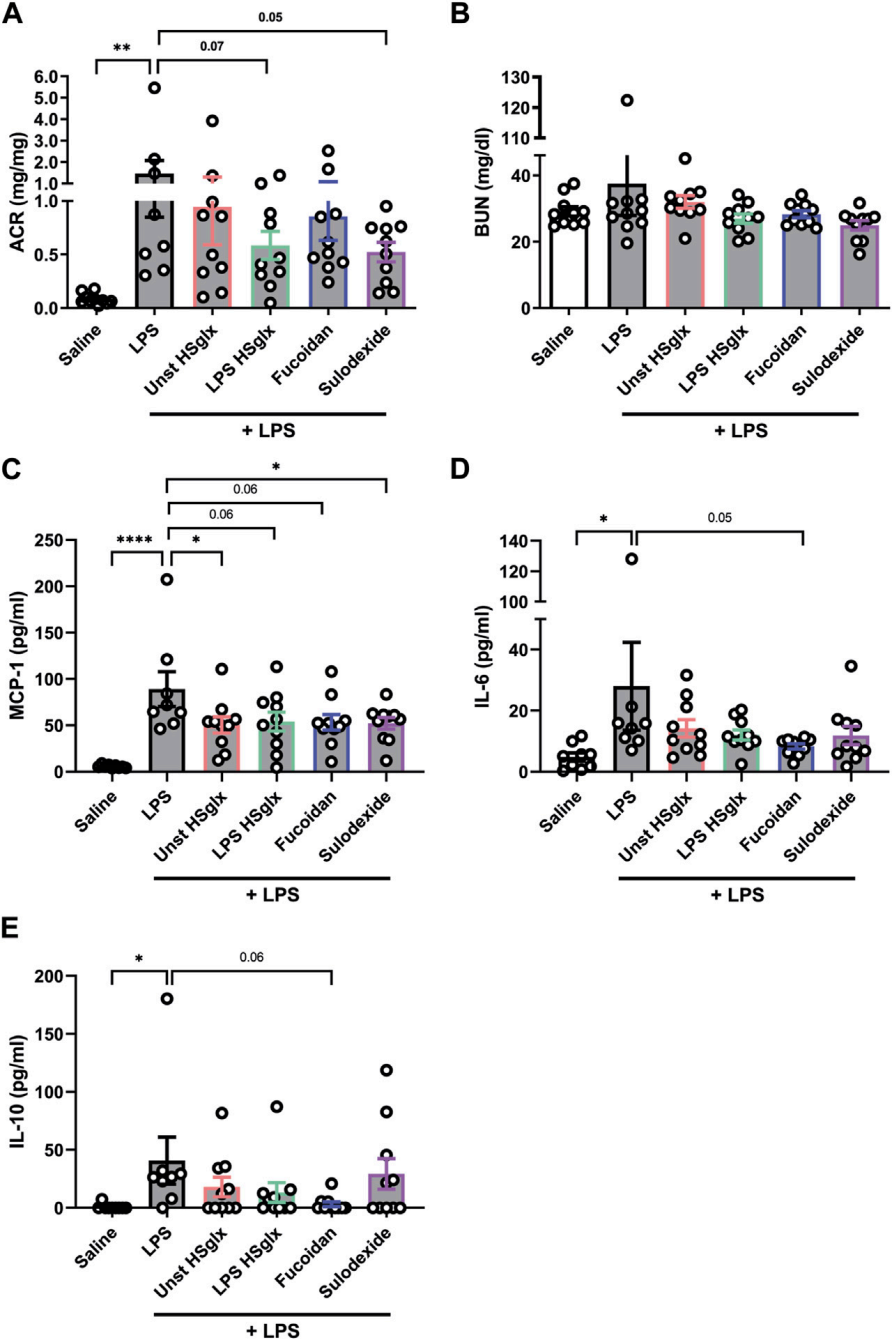
Albuminuria and plasma cytokine levels in mice with LPS-induced glomerulonephritis are in part normalized by treatment with unstimulated HS_glx_, LPS HS_glx_, fucoidan or sulodexide in mice. **(A)**. Urinary albumin/creatinine ratio and **(B)**. Renal function, as measured by BUN of mice injected with LPS (48 h) and treated with unstimulated HS_glx_, LPS HS_glx_, fucoidan or sulodexide. **(C)**. MCP-1, **(D)**. IL-6, and **(E)**. IL-10 plasma protein levels of mice injected with LPS (48 h) and treated with unstimulated HS_glx_, LPS HS_glx_, fucoidan or sulodexide. Data are expressed as mean ± SEM. *p < 0.05, **p < 0.01, ****p < 0.0001. n ≥ 8. Unstimulated HS_glx_, HS extracted from unstimulated endothelial glycocalyx; LPS HS_glx_, HS extracted from LPS stimulated endothelial glycocalyx; ACR, urinary albumin/creatinine ratio; BUN, blood urea nitrogen plasma level; MCP-1, monocyte chemoattractant protein-1.

Since LPS HS_glx_ is extracted from endothelial cells under inflammatory conditions, it is likely that LPS HS_glx_ is enriched in pro-inflammatory HS, which fuels the hypothesis that LPS HS_glx_ might be more effective for the treatment of glomerulonephritis compared to unstimulated HS_glx_. Indeed, LPS HS_glx_ attenuated proteinuria almost significantly (*p* = 0.07) in mice with LPS-induced glomerulonephritis, whereas unstimulated HS_glx_ was less potent. Sulodexide tended to reduce (*p* = 0.05) proteinuria in mice with LPS-induced glomerulonephritis as well while no clear effects were observed for fucoidan (Figure 1A). Notably, the LPS-induced glomerulonephritis model did not affect kidney function as measured by blood urea levels (Figure 1B) and plasma creatinine levels (data not shown).

### Treatment with GAGs or fucoidan affects cytokine expression levels in experimental glomerulonephritis

Since HS plays a predominant role in inflammation and cytokine increase is a main feature in LPS-induced glomerulonephritis, we investigated if cytokine expression was altered in the mice treated with unstimulated HS_glx_, LPS HS_glx_, fucoidan or sulodexide compared to LPS-injected mice that did not receive treatment. MCP-1, IL-6 and IL-10 plasma levels showed a significant increase in mice with LPS-induced glomerulonephritis compared to control mice (Figures 1B–D). On the contrary, no significant differences in plasma levels were detected between control mice and mice with LPS-induced glomerulonephritis for TNF-α, IL-23, IL-17A, IL-27, IL-12, IL-1α, IL-1β, IFN-γ, IFN-β, and GM-CSF (Supplementary Figures S1, S2).

**FIGURE 2:**
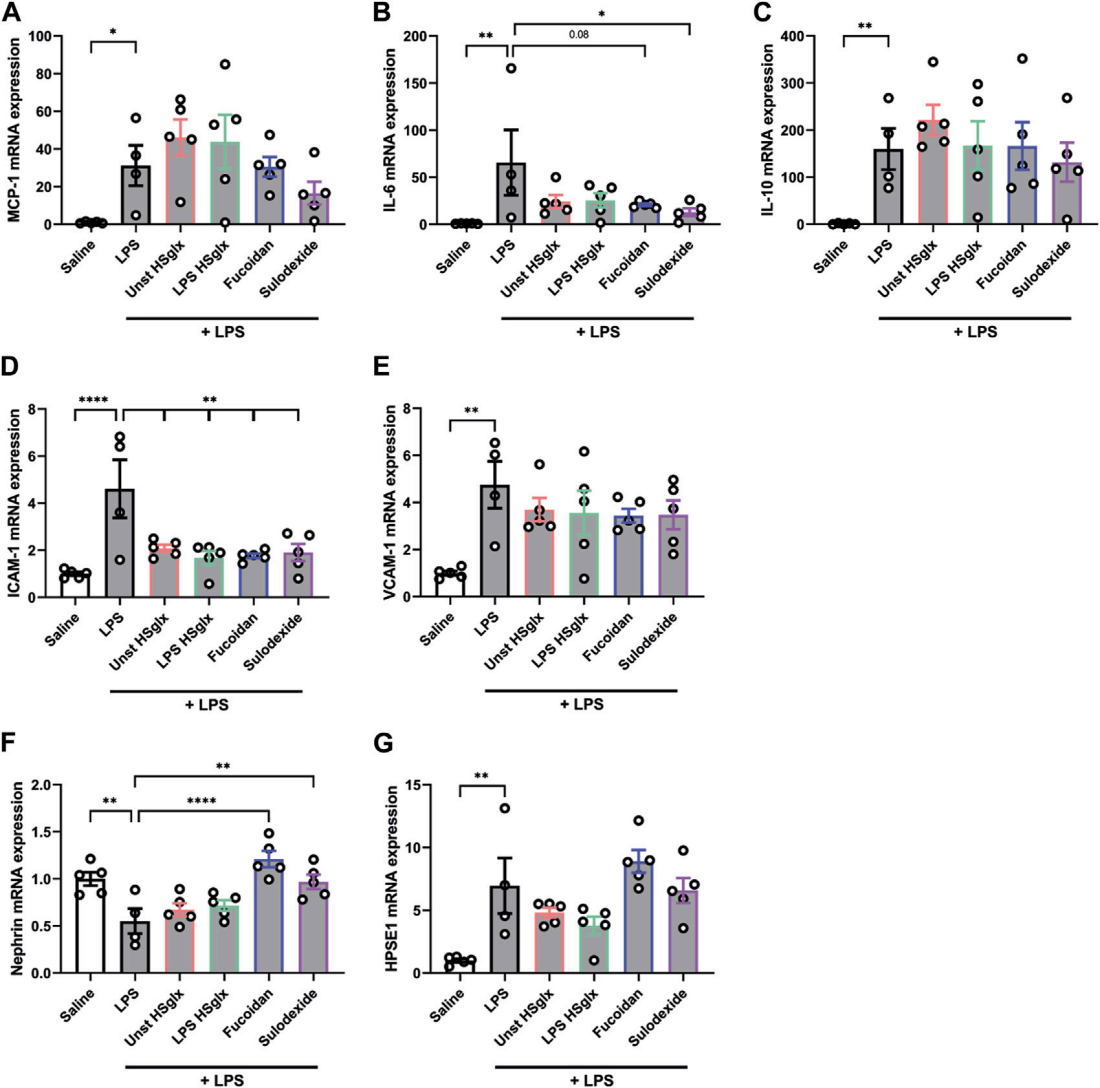
Cortical mRNA expression levels of IL-6, ICAM-1 and Nephrin in mice with LPS-induced glomerulonephritis are affected by treatment with unstimulated HS_glx_, LPS HS_glx_, fucoidan or sulodexide. **(A)**. MCP-1, **(B)**. IL-6, **(C)**. IL-10, **(D)**. ICAM-1, **(E)**. VCAM-1, **(F)**. Nephrin, and **(G)**. HPSE1 cortical mRNA expression of mice injected with LPS (48 h) and treated with unstimulated HS_glx_, LPS HS_glx_, fucoidan or sulodexide. Data are expressed as mean ± SEM. *p < 0.05, **p < 0.01, ****p < 0.0001. n ≥ 4. Unstimulated HS_glx_, HS extracted from unstimulated endothelial glycocalyx; LPS HS_glx_, HS extracted from LPS stimulated endothelial glycocalyx; MCP-1, monocyte chemoattractant protein-1; ICAM-1, intercellular adhesion molecule 1; VCAM-1, vascular cell adhesion molecule-1; HPSE1, heparanase.

Notably, MCP-1 plasma levels tended to be normalized by all 4 treatment conditions compared to mice with LPS-induced glomerulonephritis without further treatment (Figure 1C), which was not reflected in the cortical mRNA expression level of MCP-1 (Figure 2A). IL-6 plasma levels (Figure 1D) in mice with LPS-induced glomerulonephritis treated with fucoidan were near-significantly (*p* = 0.05) decreased, whereas treatment with unstimulated HS_glx_, LPS HS_glx_ or sulodexide showed a trend to decrease plasma IL-6 levels, which was in part mirrored in the cortical mRNA expression levels for IL-6 (Figure 2B). IL-10 plasma levels in mice with LPS-induced glomerulonephritis tended to decrease for all 4 treatments (Figure 1E) even though no differences were detected at the cortical mRNA levels for IL-10 (Figure 2C). Notably, the TNF-α mRNA expression level in the renal cortex did not significantly increase in mice with LPS-induced glomerulonephritis, and was not further affected by any of the treatments (Supplementary Figure S3A).

The endothelial nitric oxide synthase (eNOS) was previously shown to be involved in LPS-induced cytokine increase (Gross et al., 2015). In our study, the cortical eNOS mRNA expression did not significantly differ between mice with LPS-induced glomerulonephritis and control mice and was also not further affected by any of the GAG/fucoidan treatments (Supplementary Figure S3C).

The results of our *in vivo* experiments collectively indicate that treatment of LPS-induced glomerulonephritis with GAGs or fucoidan partially affect cytokine expression levels in plasma and kidney cortex.

### Treatment with GAGs or fucoidan affects endothelial expression of cell adhesion molecules in experimental glomerulonephritis

Intercellular adhesion molecule 1 (ICAM-1) and vascular cell adhesion molecule 1 (VCAM-1) are endothelial adhesion molecules of the immunoglobulin superfamily with a critical role in leukocyte trafficking on endothelial cells in various inflammatory diseases (Brady, 1994; Hill et al., 1994). Since glomerular influx of immune cells plays a critical role in the development of some glomerular diseases (Duffield, 2010), the mRNA expression of endothelial leukocyte adhesion molecules ICAM-1 and VCAM-1 in the renal cortex was measured. Both ICAM-1 (Figure 2D) and VCAM-1 (Figure 2E) cortical mRNA expression showed a significant increase in LPS-induced glomerulonephritis compared to control mice. Unstimulated HS_glx_, LPS HS_glx_, fucoidan or sulodexide significantly reduced the LPS-induced cortical ICAM-1 mRNA expression although none of the treatments reduced the LPS-induced cortical VCAM-1 mRNA expression (Figures 2D, E).

**FIGURE 3:**
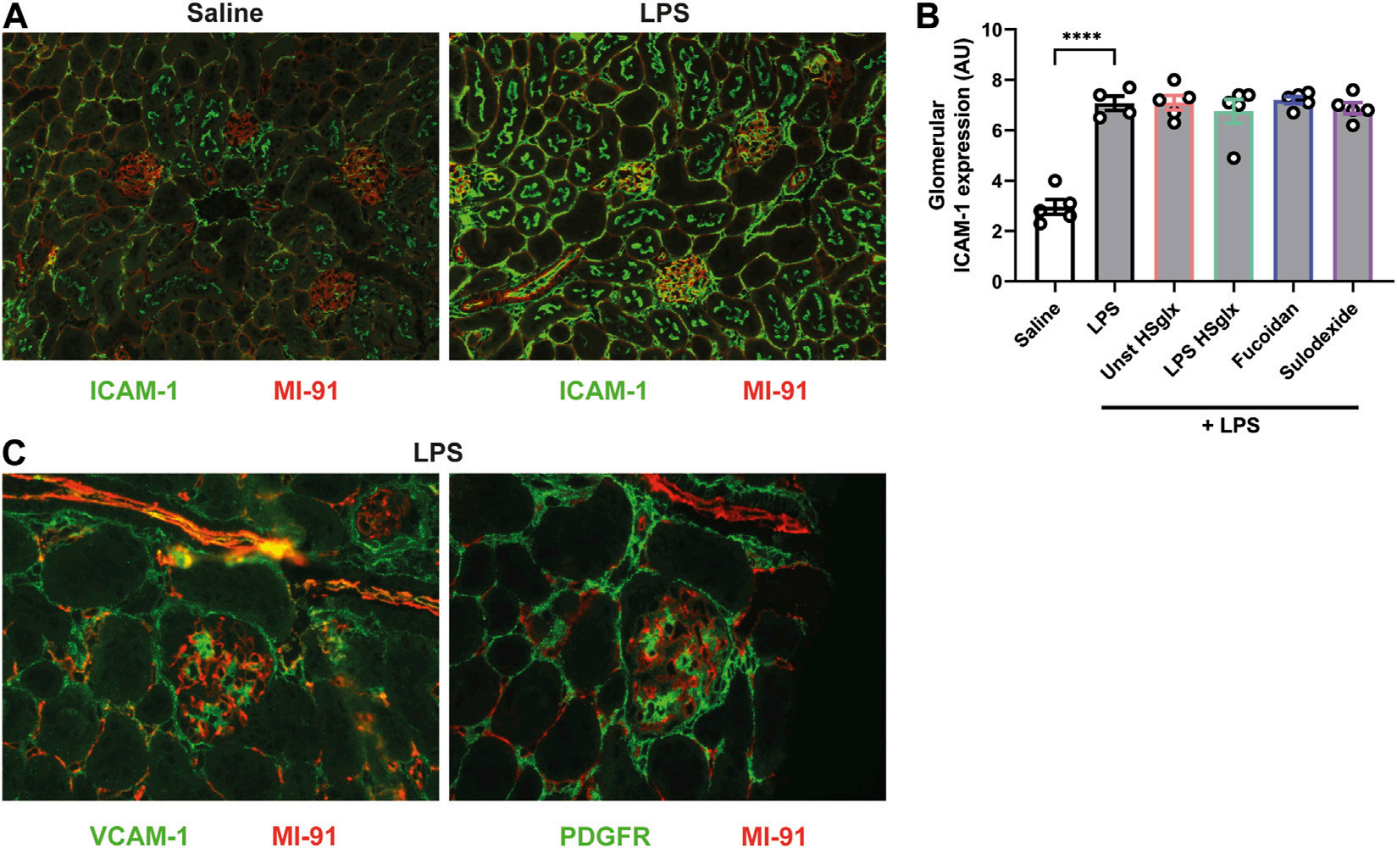
Endothelial ICAM-1 expression is increased in LPS-induced glomerulonephritis and is not affected by treatment with unstimulated HS_glx_, LPS HS_glx_, fucoidan or sulodexide. **(A)**. Expression of ICAM-1 in control kidneys and kidneys of mice with LPS-induced glomerulonephritis combined with agrin staining to visualize the glomerular basement membrane **(B)**. Semiquantitative analysis of the glomerular expression of ICAM-1 in control mice and mice with LPS induced glomerulonephritis, untreated and treated with unstimulated HS_glx_, LPS HS_glx_, fucoidan or sulodexide. **(C)**. Expression of VCAM-1 and the mesangial cell marker PDGFR shows that VCAM-1 is upregulated in mesangial cells in LPS-induced glomerulonephritis. IF staining is presented on subsequent kidney sections, in which the same glomerulus was imaged because the antibodies for VCAM-1 and PDGFR could not be combined on a single kidney section. Data are expressed as mean ± SEM. ****p < 0.0001. n ≥ 4. Unstimulated HS_glx_, HS extracted from unstimulated endothelial glycocalyx; LPS HS_glx_, HS extracted from LPS stimulated endothelial glycocalyx; ICAM-1, intercellular adhesion molecule 1; VCAM-1, vascular cell adhesion molecule-1; PDGFR, platelet-derived growth factor receptor; AU, arbitrary units.

Subsequently, the ICAM-1 and VCAM-1 protein localization and expression levels were determined by immunofluorescence (IF) staining on kidney sections, in which agrin staining was used to visualize the GBM. Both glomerular ICAM-1 (Figures 3A, B) and VCAM-1 (Figure 3C) protein expression were increased in mice with LPS-induced glomerulonephritis compared to control mice. Increased glomerular ICAM-1 expression colocalized with the endothelial marker CD31 (Supplementary Figure S5A), thereby suggesting activation of the glomerular endothelium. ICAM-1 expression also appeared increased in tubular apical membranes and peritubular capillaries (Figure 3A). Nevertheless, glomerular ICAM-1 protein expression in mice with LPS-induced glomerulonephritis mice was not affected by treatment with GAGs or Fucoidan (Figure 3B). VCAM-1 expression did not colocalize with the endothelial cell marker CD31 upon LPS stimulation (Supplementary Figure S5B). In fact, the increased VCAM-1 expression appears to colocalize with the mesangial marker platelet-derived growth factor receptor (PDGFR) (Figure 3C).

### Fucoidan and sulodexide prevent podocyte damage in experimental glomerulonephritis

Nephrin is expressed at the podocyte intercellular junction in the glomerulus and is a key component of the podocyte slit diaphragm, which is crucial to glomerular filtration. Previous studies have shown decreased nephrin expression in various human proteinuric kidney diseases as well as animal models of glomerular disease (Luimula et al., 2000; Verma et al., 2018). Therefore, we were interested if the LPS-induced decrease in nephrin expression in the renal cortex could be reversed by treatment with unstimulated HS_glx_, LPS HS_glx_, fucoidan, or sulodexide. Unstimulated HS_glx_ and LPS HS_glx_ showed a slight increase in cortical nephrin expression, whereas both fucoidan or sulodexide significantly attenuated the LPS-induced decrease in cortical nephrin expression (Figure 2F). Notably, the cortical mRNA expression of desmin, which is a podocyte activation and damage marker, was not affected by LPS-induced glomerulonephritis (Supplementary Figure S3B). These results suggest that in particular fucoidan and sulodexide seem to protect LPS-induced podocyte damage.

### Treatment with unstimulated HS_glx_, fucoidan or sulodexide reduce renal influx of immune cells in experimental glomerulonephritis

**FIGURE 4:**
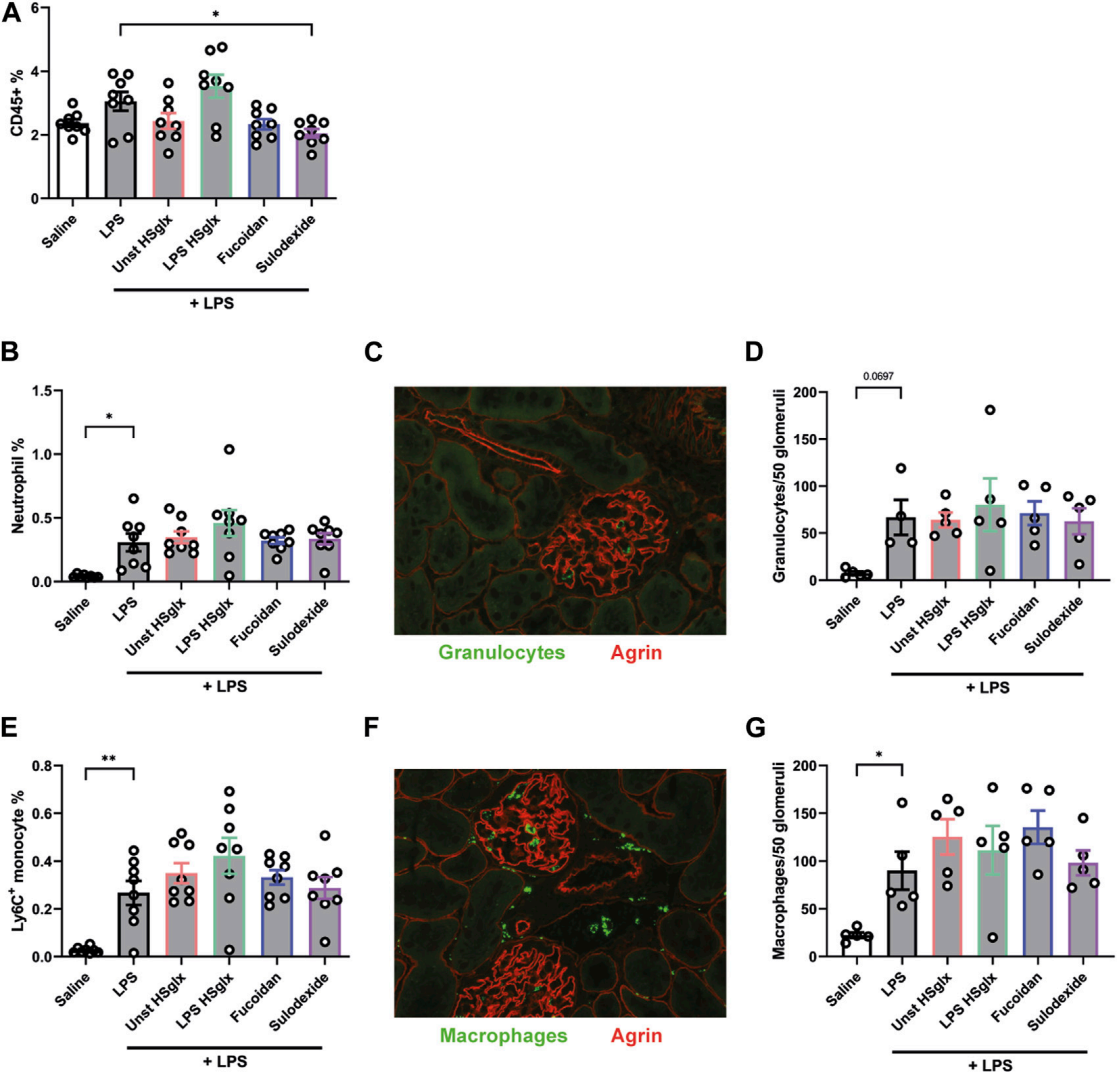
Treatment with unstimulated HS_glx_, fucoidan or sulodexide resulted in a decreased renal influx of immune cells in LPS-induced glomerulonephritis. Quantitative analysis of the influx of immune cells, specifically granulocytes and monocytes using flow cytometry and immunofluorescence. **(A)**. CD45^+^ leukocyte cell percentage of all gated cells using flow cytometry analysis. **(B)**. Neutrophil as percentage of all gated cells using flow cytometry analysis. **(C)**. Glomerular granulocyte influx as stained for with the marker Gr-1 in immunofluorescence. **(D)**. Quantitative analysis of the glomerular influx of granulocytes in mice injected with LPS and treated with unstimulated HS_glx_, LPS HS_glx_, fucoidan or sulodexide. **(E)**. Monocyte Ly6C^high^ cell percentage of all gated cells using flow cytometry analysis. **(F)**. Glomerular macrophage influx as stained for with the marker CD68 in immunofluorescence. **(G)**. Quantitative analysis of the glomerular influx of macrophages (CD68) in mice with LPS-induced glomerulonephritis, untreated and treated with unstimulated HS_glx_, LPS HS_glx_, fucoidan or sulodexide. Data are expressed as mean ± SEM. *p < 0.05, **p < 0.01. n ≥ 4. Unstimulated HS_glx_, HS extracted from unstimulated endothelial glycocalyx; LPS HS_glx_, HS extracted from LPS stimulated endothelial glycocalyx; Gr-1, granulocyte-differentiation antigen; Ly6C, lymphocyte antigen 6C.

The influx of immune cells in the kidney was determined since immune cells play a prime role in the development of glomerulonephritis. The influx of all CD45^+^, a versatile marker for all leukocytes, immune cells was increased in kidneys of LPS-induced glomerulonephritis mice compared to control mice and tended to be decreased by treatment with unstimulated HS_glx_ and fucoidan, whereas treatment with sulodexide resulted in a significantly decreased influx (Figure 4A).

The influx of neutrophils (Figure 4B) and Ly6C^+^ monocytes (Figure 4E) was increased in the kidneys of mice with LPS-induced glomerulonephritis but remained unaffected by treatment with unstimulated HS_glx_, LPS HS_glx_, fucoidan or sulodexide. Notably, quantitative analysis of glomerular granulocytes (Figures 4C, D) and glomerular macrophages influx (Figures 4F, G) revealed comparable results with the aforementioned influx of immune cells into the whole kidney as measured by flow cytometry. In summary, the inability of the compounds to attenuate the ICAM-1 protein expression in experimental glomerulonephritis is mirrored in the glomerular influx of granulocytes and monocytes.

### Unstimulated HS_glx_, LPS HS_glx_, fucoidan and sulodexide all dose-dependently inhibit HPSE activity

**FIGURE 5:**
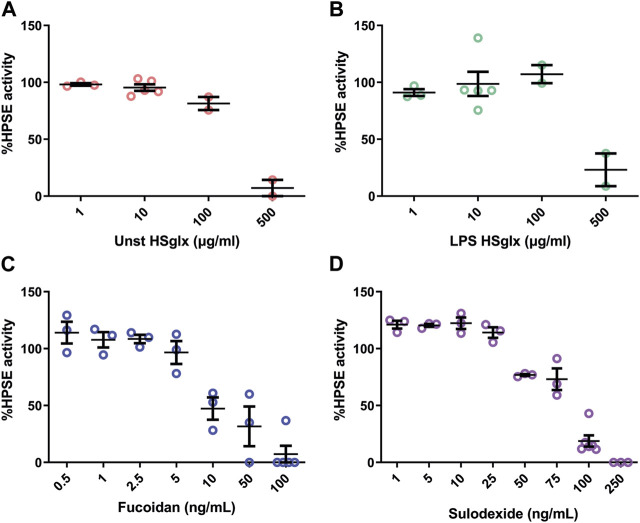
HPSE activity is dose-dependently inhibited by unstimulated HS_glx_, LPS HS_glx_, fucoidan or sulodexide. Dose response inhibition of recombinant human HPSE with **(A)** unstimulated HS_glx_ **(B)**. LPS HS_glx_, **(C)**. fucoidan and **(D)**. sulodexide. Data are expressed as mean ± SEM. Unstimulated HS_glx_, HS extracted from unstimulated endothelial glycocalyx; LPS HS_glx_, HS extracted from LPS stimulated endothelial glycocalyx; HPSE, heparanase-1.

Previous studies have shown the importance of HPSE in the development of experimental glomerulonephritis (Garsen et al., 2016). In this study, we confirmed that cortical HPSE mRNA expression was increased in the LPS-induced glomerulonephritis mice compared to controls but remained largely unaltered with the treatments (Figure 2G). Subsequently, we addressed the ability of the different compounds to inhibit HPSE activity. We observed a dose-dependent inhibition of HPSE activity *in vitro* (Figure 5) for all compounds, which is in line with previous reports that proved the HPSE inhibitory capacity of HS and sulodexide (Masola et al., 2012; Farrugia et al., 2018). Notably, our data proved direct inhibition of heparanase activity by fucoidan, which was not reported previously, and fucoidan even showed highest potency in HPSE activity inhibition followed by sulodexide, unstimulated HS_glx_ and LPS HS_glx_. These results suggest that inhibition of HPSE activity might contribute to the observed beneficial effects of GAGs and fucoidan in experimental glomerulonephritis.

### Pre-treatment with fucoidan reduces LPS-induced expression of ICAM-1 and IL-6 in mGEnC-1, but not in PBMCs

**FIGURE 6:**
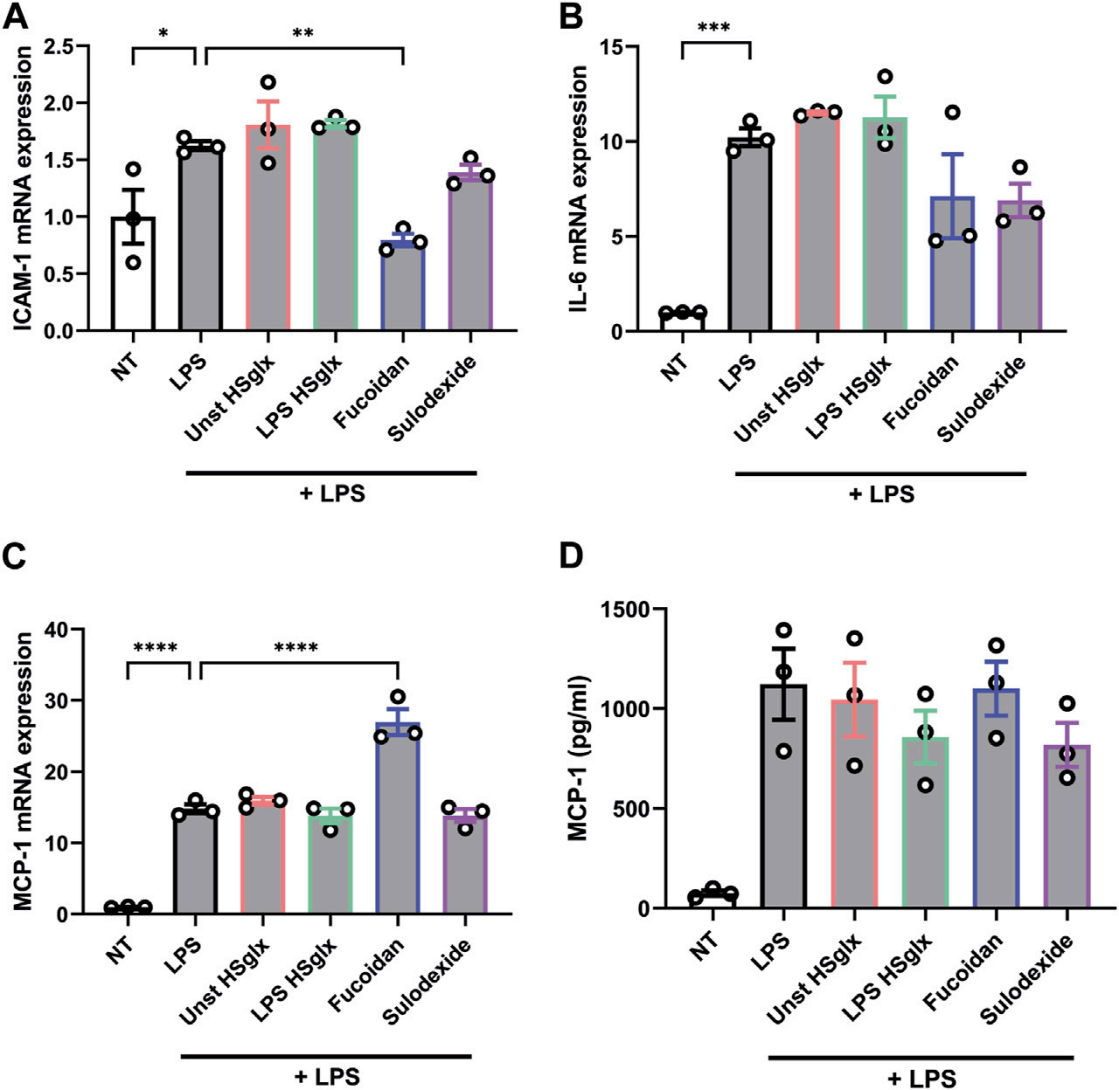
LPS-induced ICAM-1 mRNA expression in cultured mouse glomerular endothelial cells is normalized by pre-treatment with fucoidan. qPCR analysis of **(A)**. ICAM-1, **(B)**. IL-6, **(C)**. MCP-1 mRNA expression and protein secretion levels of **(D)**. MCP-1 measured in culture supernatant by ELISA of mouse glomerular endothelial cells pre-treated with 10 μg/mL unstimulated HS_glx_, LPS HS_glx_, fucoidan or sulodexide for 1 h and subsequently stimulated with 100 ng/mL LPS for 24 h. Data are expressed as mean ± SEM. *p < 0.05, **p < 0.01, ***p < 0.001, ****p < 0.0001. n ≥ 3. Unstimulated HS_glx_, HS extracted from unstimulated endothelial glycocalyx; LPS HS_glx_, HS extracted from LPS stimulated endothelial glycocalyx; ICAM-1, intercellular adhesion molecule 1; MCP-1, monocyte chemoattractant protein-1.

**FIGURE 7:**
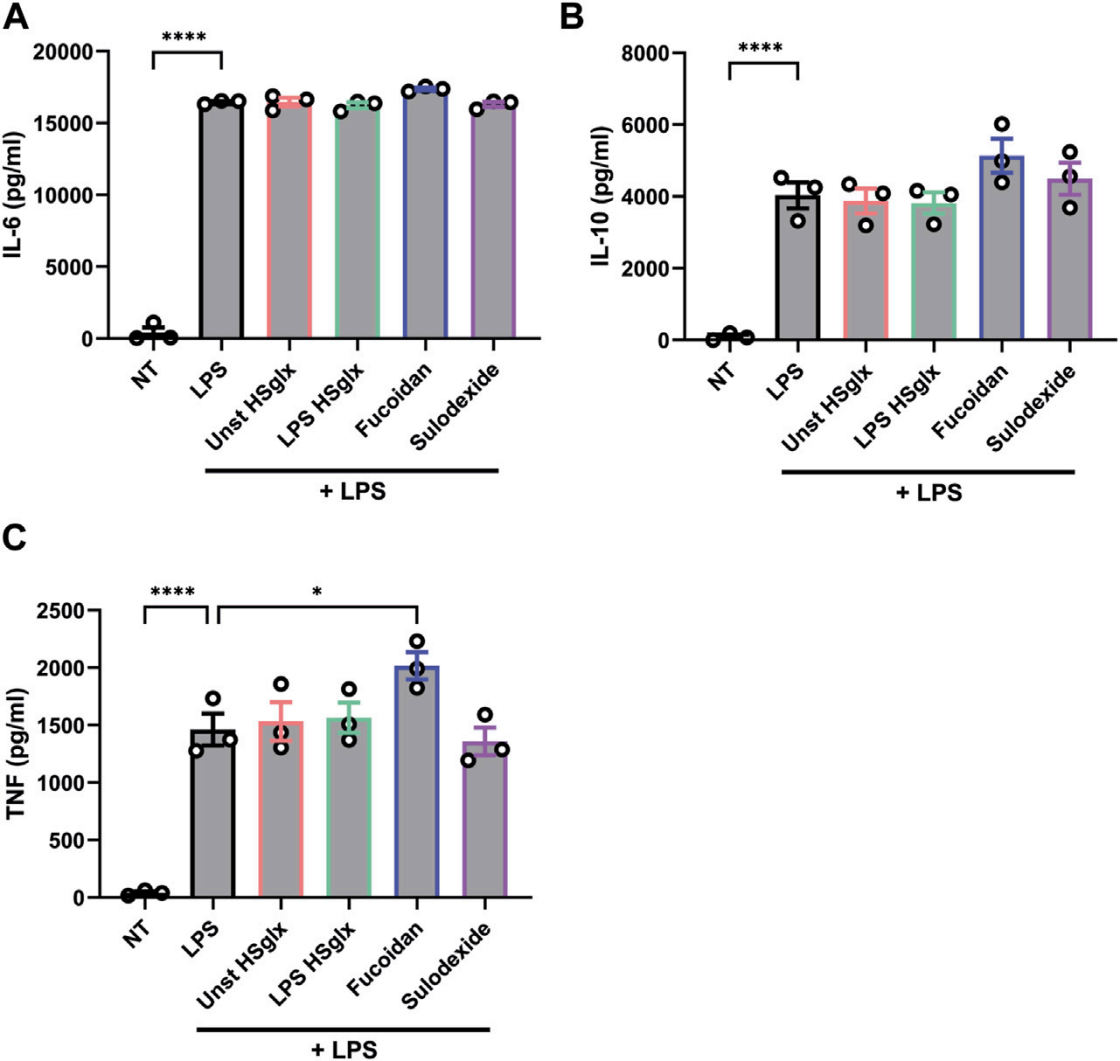
LPS-induced IL-6, IL-10 and TNF protein secretion is not affected by pre-treatment with unstimulated HS_glx_, LPS HS_glx_, fucoidan or sulodexide in human peripheral blood mononuclear cells. Protein secretion levels of **(A)**. IL-6, **(B)**. IL-10 and **(C)**. TNF measured in culture supernatant of human peripheral blood mononuclear cells pre-treated with 10 μg/mL unstimulated HS_glx_, LPS HS_glx_, fucoidan or sulodexide for 1 h and subsequently stimulated with 10 ng/mL LPS for 24 h. Data are expressed as mean ± SEM. *p < 0.05, ****p < 0.0001. n ≥ 3. Unstimulated HS_glx_, HS extracted from unstimulated endothelial glycocalyx; LPS HS_glx_, HS extracted from LPS stimulated endothelial glycocalyx.

The observed effects of unstimulated HS_glx_, LPS HS_glx_, fucoidan and sulodexide on cytokine mRNA and protein levels and ICAM-1 mRNA levels in LPS-induced glomerulonephritis can be attributed to both glomerular cells and immune cells. It is important to gain a better understanding about which cells are targeted in what manner by the treatments. Therefore, both mouse glomerular endothelial cells (mGEnC-1) and human peripheral blood mononuclear cells (PBMC) were pre-treated with unstimulated HS_glx_, LPS HS_glx_, fucoidan or sulodexide and subsequently stimulated with LPS. Both unstimulated HS_glx_ and LPS HS_glx_ did not affect the LPS-induced mRNA expression of ICAM-1 (Figure 6A), IL-6 (Figure 6B), or MCP-1 (Figure 6C) nor the protein secretion of MCP-1 in mGEnC-1 activated with LPS (Figure 6D). Sulodexide showed a decreased trend of ICAM-1 (Figure 6A) and IL-6 (Figure 6B) mRNA expression levels (Figure 6B) while no alterations were observed for MCP-1 expression levels (Figures 6C, D). Fucoidan significantly attenuated LPS-induced mGEnC-1 mRNA expression of ICAM-1 (Figure 6A) and showed a decreased trend of IL-6 mRNA expression (Figure 6B). Although fucoidan increased the mGEnC-1 mRNA expression of MCP-1 (Figure 6C), no effect was observed on MCP-1 protein secretion (Figure 6D). In addition, the PBMC protein secretion of IL-6, IL-10 and TNF-α was not attenuated by the addition of GAGs or fucoidan (Figures 7A–C). Taken together, pre-treatment with fucoidan was able to normalize the ICAM-1 and IL-6 expression of mGEnC-1, but not PBMCs, exposed to LPS.

## Discussion

GAG-based therapeutics targeting the glomerular influx of leukocytes are a newly emerging class of drugs for the treatment of a wide range of inflammatory glomerular diseases (Lindahl and Kjellen, 2013; Maciej-Hulme et al., 2018; Muralidar et al., 2021). The current study evaluated the beneficial effects of four HS(-related) compounds in experimental glomerulonephritis. We showed that treatment of mice with LPS HS_glx_ or sulodexide near-significantly attenuated LPS-induced proteinuria. In addition, unstimulated HS_glx_, LPS HS_glx_, fucoidan and sulodexide all normalized plasma MCP-1 levels and cortical ICAM-1 mRNA expression whereas no decrease in renal influx of macrophages and granulocytes was observed. Furthermore, fucoidan and sulodexide reversed cortical IL-6 and nephrin mRNA expression. Collectively, we were able to show that unstimulated HS_glx_, LPS HS_glx_, fucoidan and sulodexide all have a potentially protective effect in experimental glomerulonephritis.

HS and diversity in HS domains on (glomerular) endothelium are known to play a crucial role in inflammation (Rops et al., 2004a). Although limited information has been gathered on the chemical structure of pro-inflammatory HS domains, N- and 6-O-sulfation are important for leukocyte binding to the glomerular endothelium (Rops et al., 2008; van Gemst et al., 2021). The importance of sulfation of HS in inflammation was demonstrated with the use of N-Deacetylase and N-Sulfotransferase 1 (NDST1) deficient endothelial cells. NDST1 deficient endothelial cells produced HS with low levels of sulfation and behave different under inflammatory conditions compared to wild-type cells, both *in vivo* and *in vitro* (Wang et al., 2005). The four different compounds that were tested in this study are all sulfated, but might all comprise slightly different sulfation due to their nature. Therefore, unstimulated HS_glx_, LPS HS_glx_, fucoidan and sulodexide were expected to have distinct protective effects in experimental glomerulonephritis.

The influx of immune cells plays a key role in the development of glomerulonephritis. However, we only observed a decrease of kidney influx for all CD45^+^ cells for unstimulated HS_glx_, fucoidan and sulodexide, whereas none of the treatments reduced the influx of granulocytes and monocytes in LPS-induced glomerulonephritis. We recently showed decreased influx of granulocytes and macrophages by treatment with unstimulated HS_glx_ in anti-GBM induced glomerulonephritis in mice (Maciej-Hulme, van Gemst et al., recently accepted in Frontiers). Although the unaffected influx of granulocytes and monocytes by any treatment was unexpected, it is in line with the unaltered glomerular ICAM-1 protein expression in the different treatment groups compared to the untreated LPS-induced glomerulonephritis group. The decreased plasma levels of pro-inflammatory cytokines suggest that the immune cells might have been less activated even though the amount of glomerular infiltrating immune cells remained similar or that the majority of produced cytokines are secreted by cell sources other than kidney infiltrating immune cells.

We showed that unstimulated HS_glx_, LPS HS_glx_, fucoidan and sulodexide could all dose-dependently inhibit HPSE activity. Fucoidan and sulodexide were most potent in HPSE inhibition and also demonstrated more pronounced effects on LPS-induced cortical mRNA and protein expression of IL-6 compared to the HS_glx_ fractions. Notably, HPSE is known to induce cytokine release both directly and by HPSE mediated HS release. Increased HPSE activity releases the HS sequestered pro-inflammatory cytokines that can subsequently bind to their respective receptor (Goldberg et al., 2013; Gordts et al., 2014; Farrugia et al., 2018). These pro-inflammatory cytokines contribute to a disturbed glomerular endothelium and thereby albuminuria. HPSE-deficient mice show reduced cortical mRNA expression of pro-inflammatory cytokines (Garsen et al., 2016). Moreover, we recently showed that HPSE inhibition by heparanase-2 treatment resulted in attenuated glomerulonephritis accompanied with decreased cortical mRNA expression of IL-6 (Buijsers et al., 2023). Our observed effects of fucoidan or sulodexide in LPS-induced glomerulonephritis might thus partially be attributed to their HPSE-inhibiting capacity.

Additional beneficial effects of HS-mimetics in the development of inflammatory diseases were reported by others, which is in line with the reduced cytokine expression in glomerulonephritis by unstimulated HS_glx_, LPS HS_glx_, fucoidan and sulodexide in our study. For instance, LMWH has numerous anti-inflammatory properties and various mechanisms underlying the anti-inflammatory effect of LMWH have been proposed (Young, 2008; Ludwig, 2009; Mousavi et al., 2015). For example, LMWH can bind the majority of chemokines and cytokines and thereby is able to mediate the trafficking of leukocytes (Ramdin et al., 1998; Frevert et al., 2003; Buijsers et al., 2020b). The potential of LMWH as a therapeutic compound for inflammatory diseases has been supported by experimental models in bronchial asthma, ulcerative colitis, burns, ischemia-reperfusion, arthritis, and peritonitis (Wan et al., 2002; Young, 2008).

Similarly, fucoidan has been shown to have a protective effect on the kidneys by preventing fibrosis, inflammation, oxidative stress, and apoptosis (Carvalho et al., 2014; Zahan et al., 2022). The anti-inflammatory effect of fucoidan was demonstrated in recent studies, in which fucoidan significantly suppressed the secretion of pro-inflammatory mediators such as nitric oxide and the cytokines TNF-α and IL-1β upon LPS stimulation of macrophages (Jeong et al., 2017) and reduced the expression of proinflammatory cytokines including IL-6, TNF-α, and ICAM-1 in murine acute pancreatitis and streptozotocin-induced diabetes mellitus rat model (Carvalho et al., 2014; Aleissa et al., 2020; Zahan et al., 2022).

Fucoidan reduced the LPS-induced ICAM-1 and IL-6 mRNA expression of mGEnC-1, but no decrease of LPS-induced cytokine secretion was observed for PBMCs. This finding does not comply with previous studies, which demonstrated that fucoidan does affect the immune cells response. One study showed that the treatment of fucoidan reduced LPS-induced TNF-α, IL-1β, IL-6, and other pro-inflammatory cytokines in RAW264.7 mouse macrophages (Fernando et al., 2017). However, the fucoidan used in this study is extracted from Chnoospora minima whereas fucoidan from Laminaria japonica was used in our study. In addition, the dose of fucoidan at which an effect was detected, was five-to-ten times higher in the aforementioned study compared to our study. Another important difference is the use of human cells in our study compared to the murine study performed in RAW264.7 macrophage cell-line respectively. However, fucoidan was shown to reduce the responsiveness of human monocytes to LPS as well (Pozharitskaya et al., 2020).

Both cortical ICAM-1 and VCAM-1 mRNA expression were increased in LPS-induced glomerulonephritis in our study while the four treatments could only reduce the ICAM-1 mRNA expression and not the VCAM-1 mRNA expression. IF staining revealed that ICAM-1 was upregulated in the endothelial cells whereas VCAM-1 was upregulated in the mesangial cells, but not in the endothelial cells, upon LPS stimulation, which might explain the discrepancy in reduced VCAM-1 and ICAM-1 mRNA expression by the GAG treatments. Upregulation of VCAM-1 in mesangial cells due to LPS administration has been previously reported in a number of studies (Alpers et al., 1993; Khachigian et al., 1997; Lee et al., 2012). ICAM-1 protein expression was not decreased by any treatment. We hypothesize that this discrepancy between mRNA and protein expression might be due to kinetic differences in expression between the ICAM-1 mRNA and protein levels.

There are some limitations in our study. First, the LPS-induced glomerulonephritis model presented relatively mild, since there was no effect on kidney function. Second, we only used female mice in this study because female and male mice respond to LPS in different magnitude. Nevertheless, we induced experimental glomerulonephritis in other recent studies in male mice as well and no obvious discrepancies can be observed in disease manifestation between those studies and the current study (Garsen et al., 2016). Last, the route of administration of fucoidan and sulodexide can be discussed. Fucoidans and sulodexide are generally administered orally (Lauver and Lucchesi, 2006; Yung et al., 2013; Zahan et al., 2022) while we injected fucoidan or sulodexide intra-venously. However, there are some studies that administered fucoidan intra-peritoneal or sub-cutaneous (Zahan et al., 2022), and sulodexide intra-peritoneal and intra-venously (Borawski et al., 2007; Jo et al., 2014).

The *in vivo* effects of the treatments on glomerulonephritis were directed to different cell types; endothelial cells, podocytes and immune cells all seem to be involved. The observation that all treatments were able to alter cytokine and ICAM-1 mRNA expression levels *in vivo* but not *in vitro* suggest that the interplay between various cell types is crucial for the effect in experimental glomerulonephritis. Therefore, future research should focus on the interplay of these cells as can be performed in glomerulus on a chip models for instance.

In conclusion, we found that unstimulated HS_glx_, LPS HS_glx_, fucoidan and sulodexide display differential effects in experimental glomerulonephritis. These results contribute to a better understanding of the HS domains that are required to inhibit inflammatory processes and therefore take us one step closer towards defined HS oligosaccharides for the treatment of glomerular diseases.

## Data Availability

The raw data supporting the conclusion of this article will be made available by the authors, without undue reservation.

## Supplementary material

SUPPLEMENTARY FIGURE S1 No significant effects can be observed on plasma levels of TNF-α, IL-23, IL17A, IL27, and IL12 for LPS-induced glomerulonephritis in mice. **(A)**. TNF-α, **(B)**. IL-23, **(C)**. IL17A, **(D)**. IL27, and **(E)**. IL12 plasma protein levels of mice injected with LPS (48 hours) and treated with unstimulated HS_glx_, LPS HS_glx_, fucoidan or sulodexide. Data are expressed as mean ± SEM. n ≥ 8. Unstimulated HS_glx_, HS extracted from unstimulated endothelial glycocalyx; LPS HS_glx_, HS extracted from LPS stimulated endothelial glycocalyx; TNF-α, tumor necrosis factor-alfa.

SUPPLEMENTARY FIGURE S2 No significant effects can be observed on plasma levels of IL-1α, IL-1β, IFN-γ, IFN- β, and GM-CSF for LPS-induced glomerulonephritis in mice. **(A)**. IL-1α, **(B)**. IL-1β, **(C)**. IFN-γ, **(D)**. IFN-β, and **(E)**. GM-CSF plasma protein levels of mice injected with LPS (48 h) and treated with unstimulated HS_glx_, LPS HS_glx_, fucoidan or sulodexide. Data are expressed as mean ± SEM. n≥8. Unstimulated HS_glx_, HS extracted from unstimulated endothelial glycocalyx; LPS HS_glx_, HS extracted from LPS stimulated endothelial glycocalyx; IFN, interferon; GM-CSF, Granulocyte-macrophage colony-stimulating factor.

SUPPLEMENTARY FIGURE S3 Cortical mRNA expression of TNF-α is increased during LPS-induced glomerulonephritis in mice but is unaltered by unstimulated HS_glx_, LPS HS_glx_, fucoidan or sulodexide treatment whereas no effect on desmin and eNOS mRNA expression is observed in LPS-induced glomerulonephritis. **(A)**. TNF-α, **(B)**. desmin, and **(C)**. eNOS cortical mRNA expression of mice injected with LPS (48 h) and treated with unstimulated HS_glx_, LPS HS_glx_, fucoidan or sulodexide. Data are expressed as mean ± SEM. *p<0.05. n ≥ 4. Unstimulated HS_glx_, HS extracted from unstimulated endothelial glycocalyx; LPS HS_glx_, HS extracted from LPS stimulated endothelial glycocalyx; TNF-α, tumor necrosis factor-alfa; eNOS, endothelial nitric oxide synthase.

SUPPLEMENTARY FIGURE S4 Gating strategy for flow cytometry analysis. All cells were first gated to select only single cells (p5). Subsequently, live CD45^+^ cells were selected and further divided into lineage positive (NK cells, T cells, B cells, and erythrocytes) and lineage negative cells (granulocytes, monocytes, macrophages, and dendritic cells). The lineage negative cells were gated for the presence of Ly6G to isolate the neutrophils from the lineage negative population. Next, Ly6G negative cells were gated for CD11c and Ly6C to detect the monocytes.

SUPPLEMENTARY FIGURE S5 ICAM-1, but not VCAM-1, colocalizes with the endothelial cell marker CD31. **(A)**. Expression of ICAM-1 and the endothelial cell marker CD31 in control kidneys and kidneys of mice with LPS-induced glomerulonephritis. IF staining is presented on subsequent kidney sections, in which the same glomerulus was imaged because the ICAM-1 and CD31 antibodies could not be combined on a single kidney section. **(B).** Expression of VCAM-1 and CD31 in control kidneys and kidneys of mice with LPS-induced glomerulonephritis show that VCAM-1 is not upregulated in endothelial cells in LPS-induced glomerulonephritis. ICAM-1, intercellular adhesion molecule 1; VCAM-1, vascular cell adhesion molecule-1.
