# Supplementary figures and images for: Glycosaminoglycans and fucoidan have a protective effect on experimental glomerulonephritis
Source: Front Mol Biosci. 2023 Jul 5;10:1223972. doi: 10.3389/fmolb.2023.1223972 (PMC10354240; doi:10.3389/fmolb.2023.1223972)

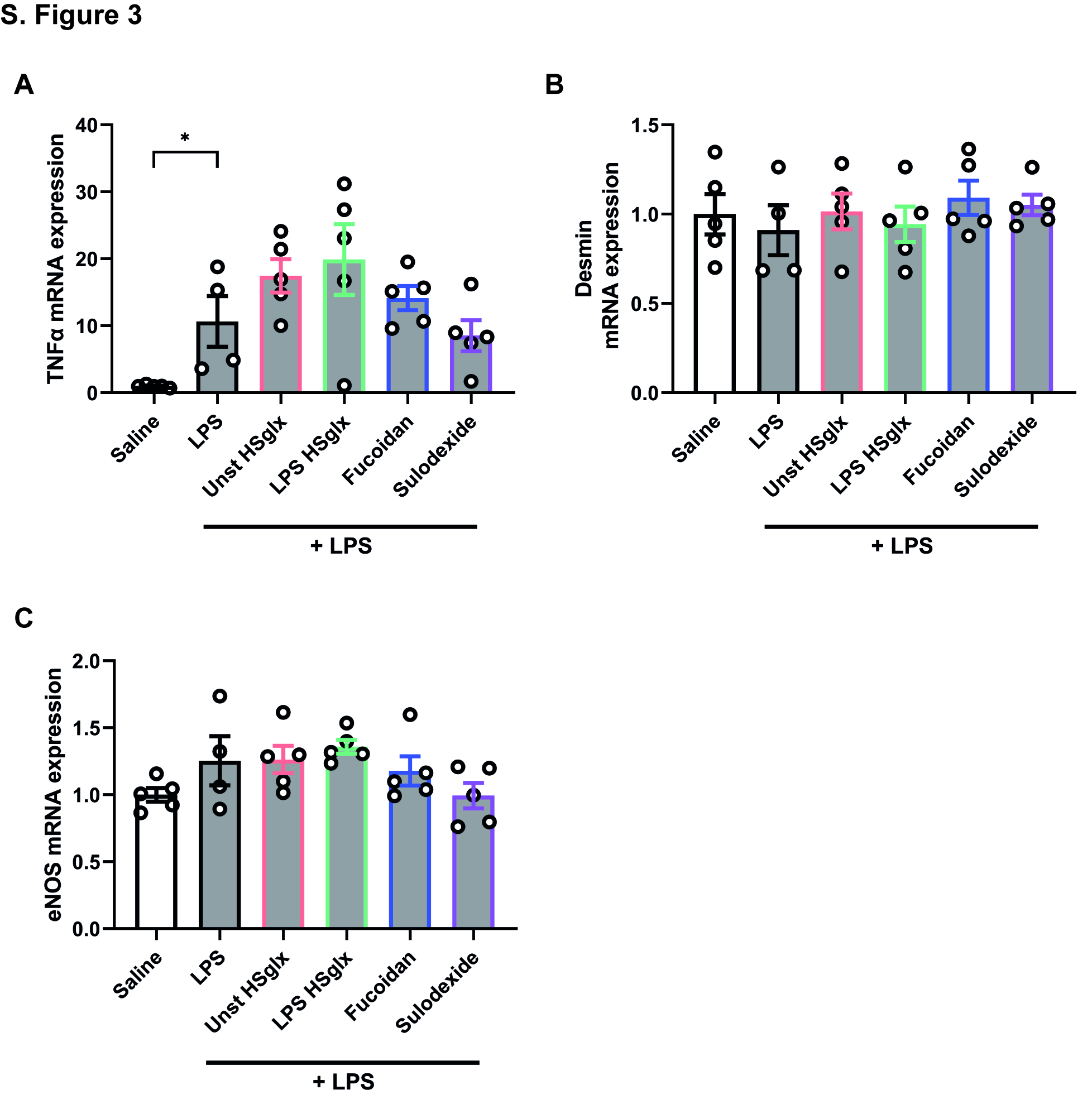

Supplement: Supplementary file 1 [file Image3.TIF]

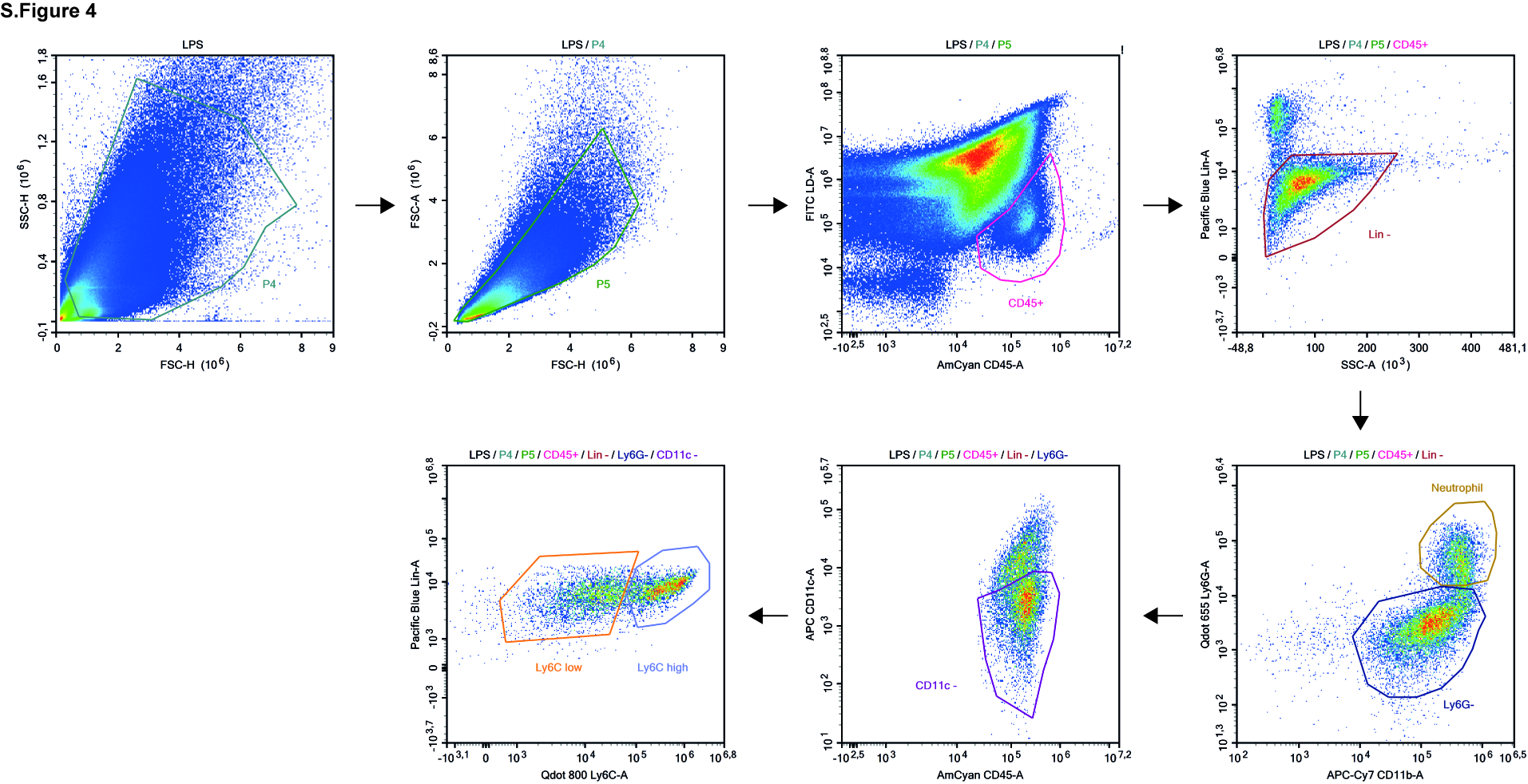

Supplement: Supplementary file 2 [file Image4.TIF]

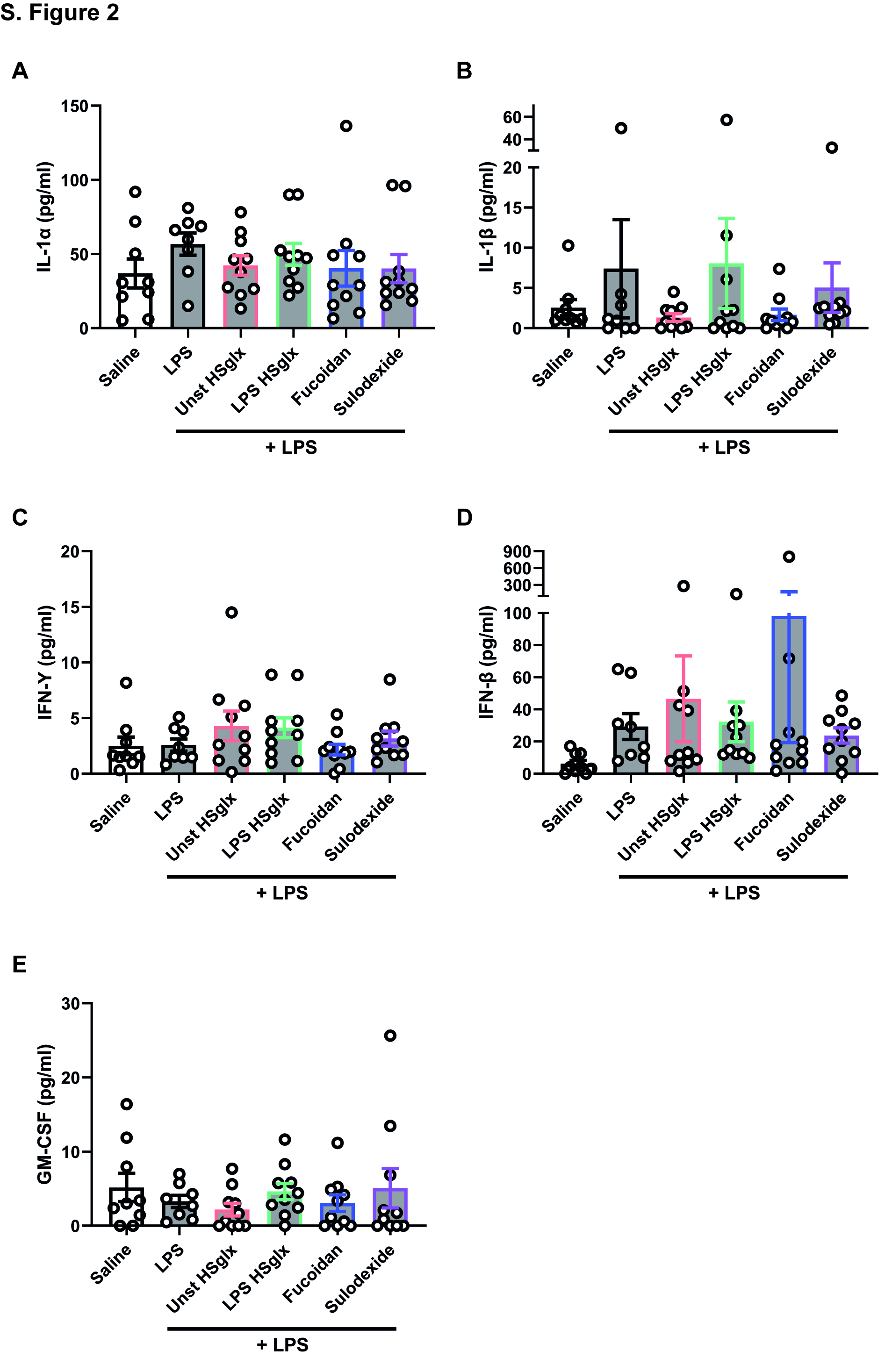

Supplement: Supplementary file 3 [file Image2.TIF]

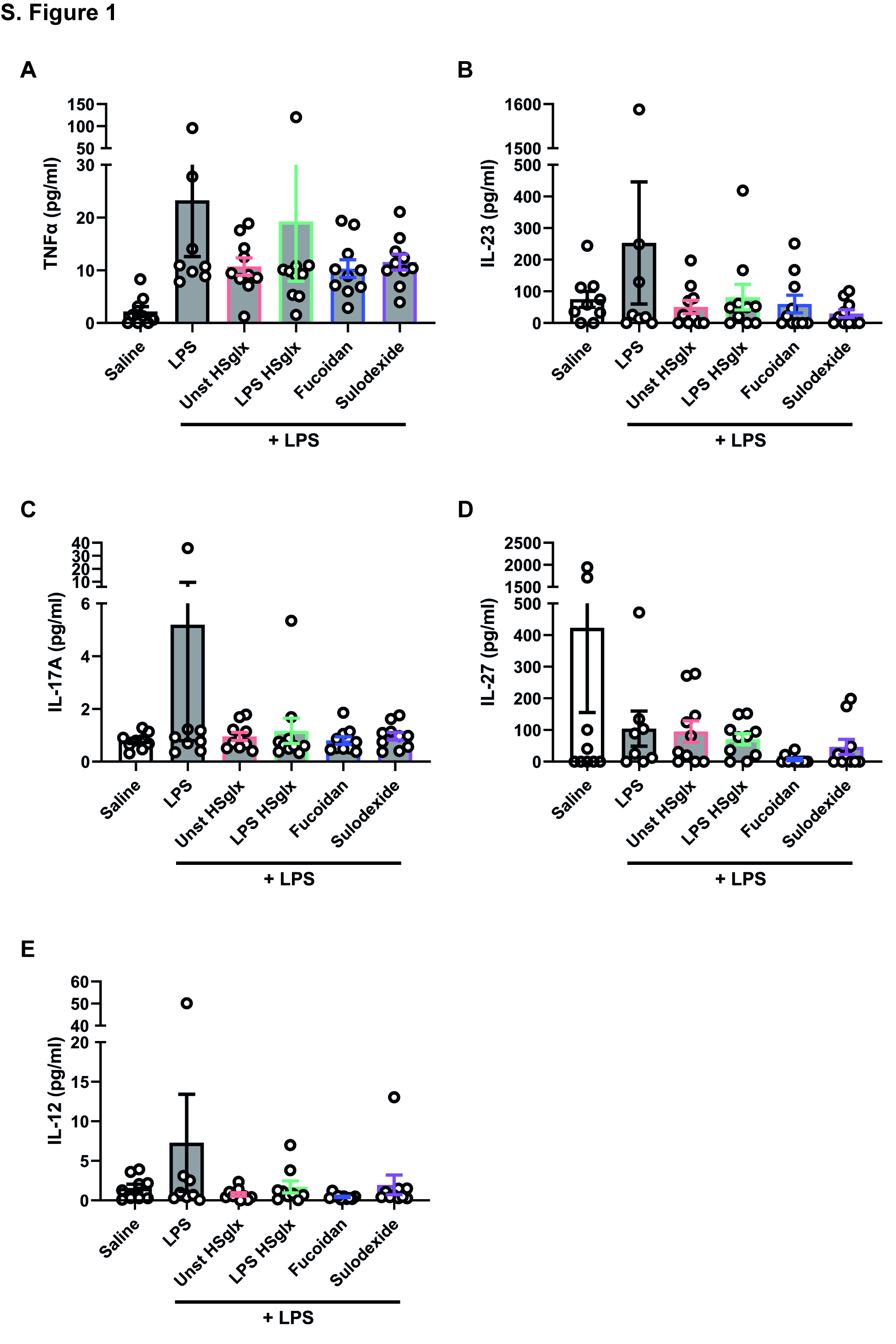

Supplement: Supplementary file 4 [file Image1.TIF]

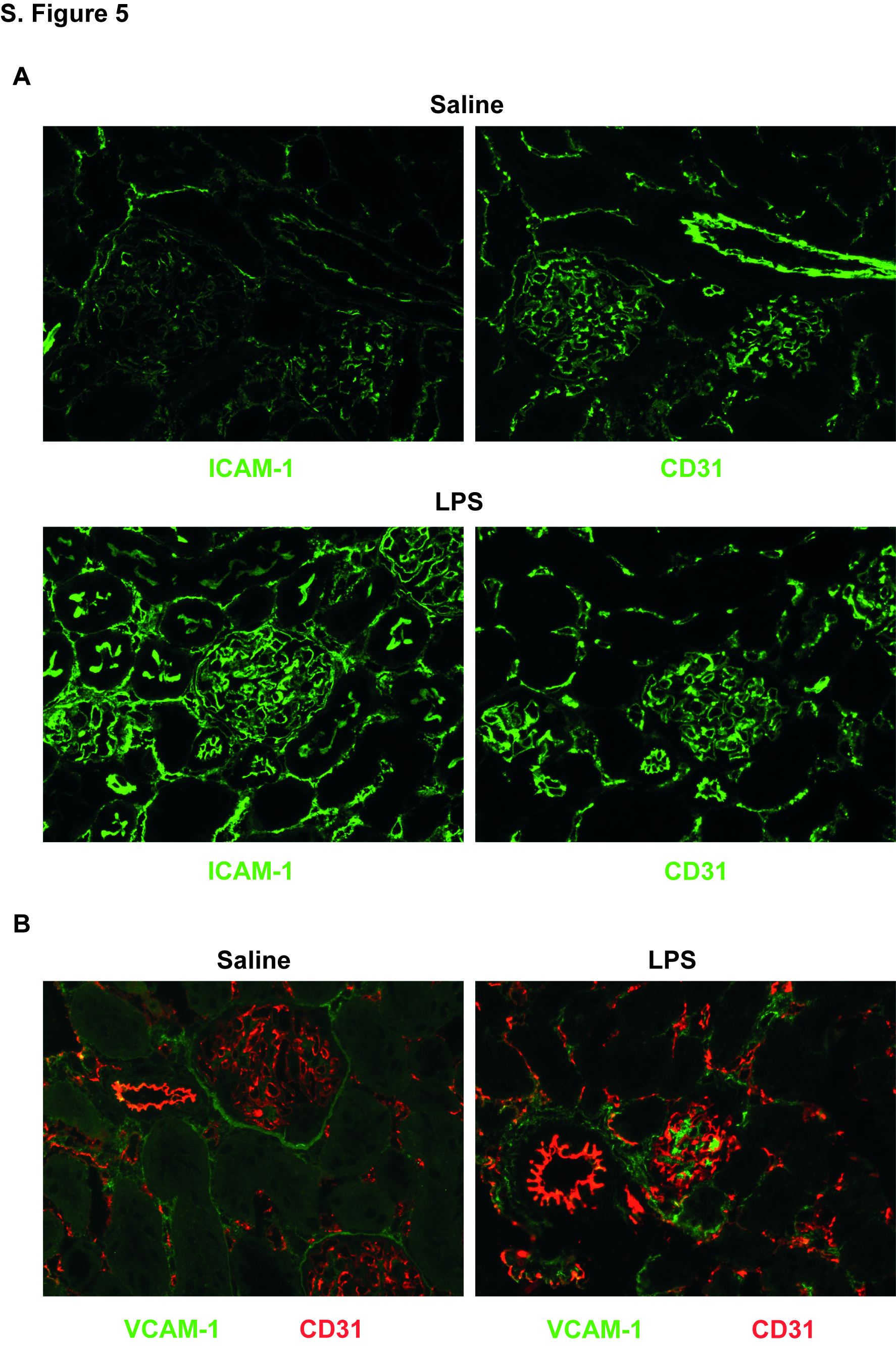

Supplement: Supplementary file 5 [file Image5.TIF]
